# Staple-line recurrence arising 10 years after functional end-to-end anastomosis for colon cancer: a case report

**DOI:** 10.1186/s40792-014-0011-3

**Published:** 2015-01-29

**Authors:** Akira Ouchi, Masahiko Asano, Keiya Aono, Tetsuya Watanabe, Shingo Oya

**Affiliations:** Department of Surgery, Chita City Hospital, 2-1 Nagai, Shinchi, Chita, Aichi 478-8640 Japan

**Keywords:** Staple-line recurrence, Colon cancer, Functional end-to-end anastomosis

## Abstract

We report a rare case of late staple-line recurrence arising 10 years after functional end-to-end anastomosis for splenic flexure colon cancer. An 80-year-old man, who underwent partial colectomy with functional end-to-end anastomosis for splenic flexure colon cancer 10 years earlier, presented with a chief complaint of anorexia. Complete blood count showed anemia, and the fecal occult blood test was positive. Lower gastrointestinal series showed an irregular defect of the splenic flexure, and colonoscopy showed an ulcerated tumor on the staple line of the primary surgery. Partial colectomy was performed, and the tumor was pathologically diagnosed as moderately differentiated tubular adenocarcinoma, resembling the pathology of primary colon cancer. This case suggests the importance of considering staple-line recurrence after functional end-to-end anastomosis for colon cancer even more than 5 years after primary surgery.

## Background

Mechanically stapled anastomosis (MSA) is a widely used technique in various digestive surgeries, and functional end-to-end anastomosis (FEEA) is often used in lower gastrointestinal surgeries. Several studies have reported that FEEA commensurates the surgical technique of surgeons and decreases anastomotic leakage, the risk of surgical site infection, and the surgery time [1,2]. FEEA is an easy and safe technique compared with conventional hand-sewn anastomosis; and in recent years, it has been used by many surgeons for reconstruction after a lower gastrointestinal surgery.

Meanwhile, it is presumed that FEEA occasionally causes staple-line recurrence, which is due to the implantation of free intraluminal cancer cells [3]. Staple-line recurrence occurs few years after primary surgery, and careful follow-up is required during this period. We report a rare case of late staple-line recurrence arising 10 years after FEEA for splenic flexure colon cancer. This case report highlights the importance of considering staple-line recurrence and a careful follow-up for patients with FEEA for colon cancer.

## Case presentation

An 80-year-old Japanese male, with a history of hypertension, had undergone partial colectomy with FEEA using autosutures (Endo GIA™, Covidien, Ireland) for splenic flexure colon cancer and distal gastrectomy with hand-sewn retrocolic Billroth-II gastrojejunostomy 10 years before. Pathological examination had revealed moderately differentiated tubular adenocarcinoma with KRAS wild-type, positive immunohistochemical staining for p53 and cdx2, negative for CD10 and MUC5AC, invading the subserosa without lymph node metastases. The cancer was resected with distal and proximal margin of each 10 cm, and no tumor cells had been identified at the surgical margins. Carcinoembryonic antigen (CEA) and carbohydrate antigen 19–9 (CA 19–9) were within the normal range. He had been well without any signs of locoregional recurrence and distant metastases. He had undergone the latest colonoscopy 7 years before and contrast-enhanced computed tomography (CECT) 5 years before.

**Figure 1:**
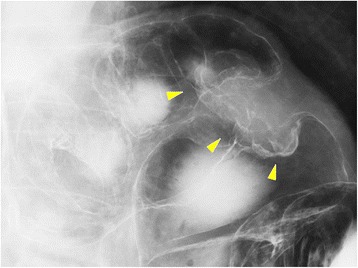
**Lower gastrointestinal series showed an irregular defect of the splenic flexure (arrows).**

**Figure 2:**
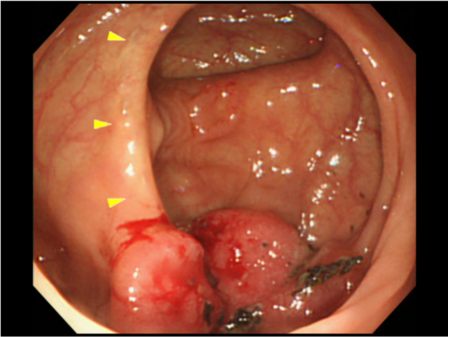
**Colonoscopy showed an ulcerated macroscopic type 2 tumor on the staple line (arrows).**

**Figure 3:**
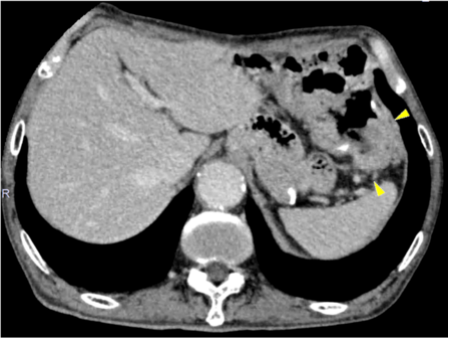
**Contrast-enhanced computed tomography (CECT) showed a splenic flexure colon tumor at the staple line (arrows).**

**Figure 4:**
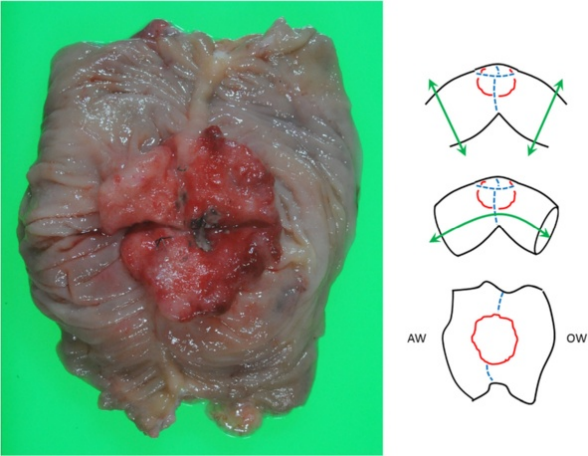
**A resected specimen contained an ulcerated macroscopic type 2 tumor destructive of crossed staple line.**

**Figure 5:**
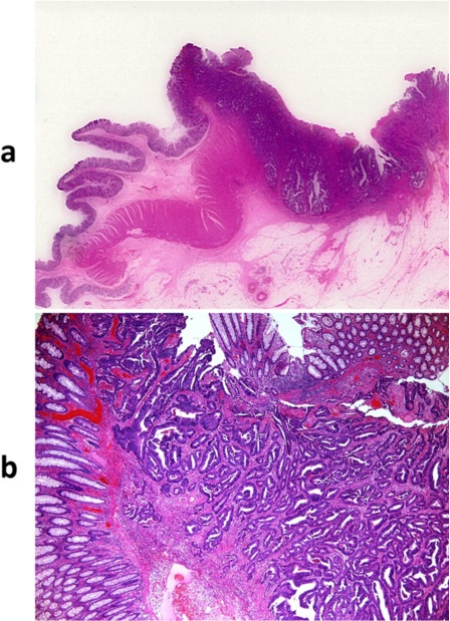
**Pathological examination revealed moderately differentiated tubular adenocarcinoma (H.E. stain; a × 4, b × 40).**

**Figure 6:**
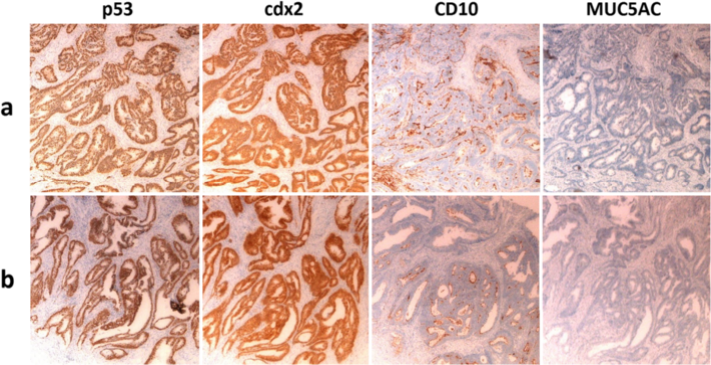
**The pathology of resected specimen (a) resembled the pathology of primary colon cancer (b).**

The patient presented to our clinic with a chief complaint of anorexia. He had slight conjunctival pallor, and a complete blood count and blood biochemistry showed a low red blood cell count (397 × 10^4^/mL) and a low hemoglobin count (12.9 g/dL). CEA and CA 19–9 were within the normal range. We performed a fecal occult blood test twice, and it was positive on both occasions. We suspected metachronous colorectal cancer and performed lower gastrointestinal series and colonoscopy for further examination. Lower gastrointestinal series showed an irregular defect of the splenic flexure near the anastomosis line of the primary surgery (Figure 1), and colonoscopy showed an ulcerated macroscopic type 2 tumor on the staple line of the primary surgery (Figure 2). Pathological examination of a biopsy specimen obtained by colonoscopy revealed moderately differentiated tubular adenocarcinoma. CECT showed a colon tumor of the splenic flexure at the staple line, but no distant metastases were found (Figure 3). The suspicion of staple-line recurrence after FEEA of the primary surgery was confirmed, and we performed open partial colectomy for radical resection. For reconstruction after partial colectomy, we performed hand-sewn end-to-end colocolostomy by the Gambee method using 3–0 Vicryl™ (Ethicon Endo-surgery; Johnson & Johnson K.K., USA). The surgery time was 159 min, and blood loss was 250 g. A resected specimen during the surgery contained an ulcerated macroscopic type 2 tumor, 4 × 4 cm in diameter, destructive of crossed staple line (Figure 4). A pathological examination revealed moderately differentiated tubular adenocarcinoma, invading the subserosa (Figure 5). The pathology of resected specimen resembled the pathology of primary colon cancer with KRAS wild-type, positive immunohistochemical staining for p53 and cdx2 and negative for CD10 and MUC5AC (Figure 6). We thus finally diagnosed the patient with staple-line recurrence, arising 10 years after FEEA of the primary surgery.

Although the patient developed symptomatic anastomotic leakage (Clavien-Dindo Grade IIIA; [4]), he improved after treatment with percutaneous drainage and was discharged from the hospital 45 days after the surgery in good health. The patient underwent a colonoscopy 12 months after the secondary surgery, and the colonoscopy showed no signs of anastomotic recurrence. He has remained well without any signs of further recurrence at the 15-month follow-up.

## Discussion

Locoregional recurrence is the most frequent recurrence in patients with resected colon cancer apart from liver metastasis and lung metastasis [5]. Locoregional recurrence is classified as either local recurrence or regional recurrence. Local recurrence is defined as tumor regrowth at anastomosis or immediately within or contiguous to the operative area, and the tumor regrowth at anastomosis is usually called anastomotic recurrence [6]. The mechanism of tumor regrowth in anastomotic recurrence is as follows: free intraluminal cancer cells of colonic origin penetrate through watertight anastomoses, implant on the anastomotic surface, and initiate tumor regrowth [3]. MSA such as functional end-to-end anastomosis or double stapling technique (DST) reportedly has a higher risk of anastomotic recurrence (in particular, staple-line recurrence) than hand-sewn anastomosis [7-9]. However, the reason for the high recurrence rate in MSA is unclear. Intraoperative intestinal irrigation reportedly reduces staple-line recurrence with DST for rectal cancer and now is widely performed [10]. In contrast, intraoperative intestinal irrigation is not often performed during FEEA for colon cancer mainly because of the challenging procedure and the high risk of surgical field contamination. Hasegawa et al. reported that surgical bowel occlusion around the tumor and intraluminal lavage can prevent or eliminate exfoliated malignant cells at anastomotic sites [11], but it is unclear whether intraoperative intestinal irrigation in FEEA for colon cancer decreases staple-line recurrence. Till date, there are no recommendations on how to decrease staple-line recurrence with FEEA for colon cancer.

Most postoperative recurrences in patients with resected colon cancer occur during the first 5 years; especially, almost all staple-line recurrences occur within the first 3 years after primary surgery [12,13]. In many countries, the follow-up period in patients with resected colon cancer is 5 years, similar to Japan [14-16]. There are few reports on late recurrence, including staple-line recurrence, of colon cancer more than 10 years after primary surgery. To the best of our knowledge, this is the first report on late staple-line recurrence arising 10 years after FEEA for colon cancer.

Colonoscopy is useful for finding staple-line recurrence after FEEA for colon cancer, and it is recommended for surveillance in patients with resected colon cancer in addition to history taking, physical examination, CEA measurement, and CECT scan in several guidelines [14-16]. For example, NCCN clinical practice guidelines for colon cancer suggest that patients with resected colon cancer should undergo colonoscopy 1 year after primary surgery and the subsequent examination is recommended 3 years from first examination, if normal, and the third examination 5 years from the second examination, if normal. Colonoscopy at this stage, however, is not performed for the detection of locoregional recurrence of primary cancer, enabling curative treatment, but it is performed for the detection of metachronous colorectal cancer [17]. Several randomized controlled trials of intensive surveillance with colonoscopy and meta-analyses of these trials have shown no survival benefit from primary cancer by performing colonoscopy at annual or shorter intervals; therefore, the goal of surveillance with colonoscopy in patients with resected colon cancer is to detect metachronous colorectal cancer at an early stage [18-23]. Routine colonoscopy is not appropriate in all patients with resected colon cancer to detect staple-line recurrence after FEEA considering the relatively low incidence of staple-line recurrence in colon cancer. To detect staple-line recurrence after FEEA, careful work-ups must be performed only on patients with a history of resected colon cancer who complain of any digestive organ symptoms.

## Conclusions

Our case suggests the possibility of late staple-line recurrence after FEEA for colon cancer even more than 5 years after primary surgery. Consideration of staple-line recurrence and careful work-up is important when patients with FEEA for colon cancer complain of any digestive organ symptoms.

## Consent

Written informed consent was obtained from the patient for publication of this case report and any accompanying images. A copy of the written consent is available for review by the Editor-in-Chief of this journal.
